# Knowledge and attitude towards blood donation among college students in Southwest Ethiopia

**DOI:** 10.11604/pamj.2021.38.249.22411

**Published:** 2021-03-09

**Authors:** Tewodros Yosef, Wondimagegn Wondimu, Melkamsew Tesfaye, Aragaw Tesfaw

**Affiliations:** 1 Department of Epidemiology and Biostatistics, School of Public Health, College of Medicine and Health Sciences, Mizan-Tepi University, Mizan-Aman, Ethiopia,; 2 Department of Nutrition and Reproductive Health, School of Public Health, College of Medicine and Health Sciences, Mizan-Tepi University, Mizan-Aman, Ethiopia,; 3 Department of Public Health, College of Health Sciences, Debre Tabor University, Debre Tabor, Ethiopia

**Keywords:** Attitude, blood donation, knowledge, college students, Ethiopia

## Abstract

**Introduction:**

blood donation (BD) is affected by several factors, among which people's knowledge and attitude are the key determinants. However, the level of knowledge and attitude towards BD in Ethiopia is not yet well studied. Therefore, this study aimed to assess the level and factors associated with knowledge and attitude towards blood donation among health science college students in Southwest Ethiopia.

**Methods:**

a cross-sectional study was conducted among 394 health science students from June 1^st^ to 15^th^ 2019. The data were collected using a structured self-administered questionnaire. The data were entered using EPI-data version 4.2.0.0 and analyzed using SPSS version 20. The correlation analysis was done to determine the association between the knowledge sum score and the attitude sum score. A binary logistic regression analysis was done to determine the association between the dependent and independent variables.

**Results:**

the proportions of good knowledge and positive attitude towards BD were 69.3%, 95% CI (64.8%-73.4%) and 58.1%, 95% CI (52.3%-63.0%) respectively. The study also found that age ≥23 years (adjusted odds ratio (AOR)=1.67, 95% CI (1.04-2.67)), having a father with primary and secondary school and above (AOR=2.24, 95% CI (1.20-4.17) and AOR=2.26, 95% CI (1.26-4.06) respectively) and ever donated blood (AOR=3.64, 95%CI (2.26-5.85)) were factors associated with good knowledge of blood donation. Being a rural resident (AOR=1.59, 95% CI (1.01-2.40)) and graduating class student (AOR=0.56, 95% CI (0.34-0.96)) were factors associated with a positive attitude towards blood donation. The knowledge-related questions´ sum score value was positively correlated with the attitude-related questions’ sum score value (r=0.30, P<0.001).

**Conclusion:**

the knowledge and attitude towards BD among the study population are a substantial deficiency. Therefore, more effort is needed to increase the level of knowledge and attitude towards BD by inculcating short training courses for these groups of population in the existing curriculum.

## Introduction

Blood transfusion is a core service within the health care system and individuals who donate their blood provide a unique contribution to the health service and survival of others [1]. Transfusion is one of the major medical interventions for the multiplicity of diseases and injuries [2]. Each year, millions of lives are saved through blood transfusion [3]. However, in most developing countries, including Ethiopia, peoples still die due to an inadequate supply of blood and blood products [3-5].

The World Health Organization advocates that 3-5% of the population should donate blood yearly [6]. Blood can only be gained from generous donors [7]. Youths, particularly medical students, constitute the core group for recruitment and retention of voluntary donor populations to ensure safe and sustainable blood transfusion [8,9]. The process of blood donation (BD) is affected by several factors, among which people´s level of knowledge and attitude are the key determinants [10].

The lack of knowledge about blood donation is a major concern reported in most studies conducted globally [11]. The lack of knowledge about blood donation and various misconceptions has resulted in a limited number of voluntary donors [12]. The greater one´s knowledge in the blood donation process, the more likely one would donate blood [11]. A study conducted by Arage *et al*. revealed that good knowledge and a positive attitude towards BD are the determinant factors for the practice of voluntary BD [4].

Several studies conducted Worldwide regarding knowledge and attitude towards blood donation revealed that 48.2% and 79.2% in Gondar [13], 40.4% and 47.4% in Ambo [9], 54% and 65.8% in Samara [5], 82.6% and 58.7% in Woliyta Sodo [7], 56.8% and 82% in Gondar [14], 43.5% and 32.9% in Harar [15] studies in Ethiopia, 66.7% and 68.7% in Saudi Arabia [16], 97.1% and 88.8% in Malaysia [17], 98% and 67% in Pakistan [18] and 15.5% and 17.69% in Iran [2] of the respondents had good knowledge and positive attitude towards blood donation respectively. The factors that affect the level of knowledge and attitude towards blood donation are varied from region to region based on deference´s in age, gender, residence, academic year, parent education and experience of blood donation [2,8-10,15,17,19,20].

Health science students are promising forces in the future practice of voluntary blood donation and also act as role models plus motivators for the community by creating awareness to increase the number of donors when they engage in the world of work [13], however, the level of knowledge and attitude among these populations has not yet been well studied in the study area. Therefore, understanding the various factors contributing to knowledge and attitude towards blood donation among health science students has paramount importance. Therefore, this study aimed to assess the level and factors associated with knowledge and attitude towards blood donation among Mizan-Aman health science college students in southwest Ethiopia.

## Methods

**Study design, setting and period:** a cross-sectional study was conducted at Mizan-Aman Health Science College (MAHSC) from June 1^st^ to 15^th^ 2019. The college is found in Mizan-Aman town, Bench Sheko zone at 585km to the southwest of Addis Ababa (the capital city of Ethiopia). There were seven departments in the college: clinical nurse, laboratory technician, health information technology, health extension workers, midwifery, pharmacy and emergency treatment. There were 2130 students from all departments, of whom 827 males and 1303 females during the study period.

**Source and study populations:** all regular health science students were the source population for this study. The study populations were randomly selected health science students.

**Sample size determination and sampling techniques:** the sample size was determined using a single population proportion formula with the input of estimated proportion of practice blood donation (21.6%) [7], 5% precision level, 95% confidence level, 10% for non-response compensation and a design effect of 1.5. The computed sample size was 429. A two-stage stratified sampling technique was used to select 429 regular students. In the college, there were seven departments and each department had three levels (level I-level III). First, students were stratified based on their departments and given a proportional sample size allocation for each department. Then, the sample size was proportionally allocated for each level within each department. Finally, the potential study participants were selected using a simple random sampling technique using the recorded list of students in each year level (batch) of the department as a sampling frame.

**Data collection instrument and procedures:** the data were collected using a structured self-administered questionnaire. The questionnaire was composed of three parts: socio-demographic factors, knowledge-related questions (10 questions), and attitude-related questions (7 questions). The questionnaire was developed by reviewing relevant literature in English. Then, it was translated to the local language and translated back to English to check the consistency by independent translators. The training was given to data collectors and supervisors concerning the objective and process of data collection for two days. There were discussions on ambiguous questions in the questionnaire and clarity was made. A pre-test was conducted on 5% of the study population in another college before data collection. After a minimal modification of the questionnaire, the data collectors distributed the questionnaires to students and they followed them while filling the questionnaires. The supervisors follow and monitor the overall data collection process to maintain the quality of the data.

**Study variables:** the dependent variables were knowledge and attitude towards blood donation. The independent variables were age, sex, place of residence, academic year, parent education and ever donated blood.

**Operational definitions:** knowledge: respondents who scored the mean value and above for knowledge-related questions were considered to have good knowledge and otherwise considered to have poor knowledge [19]. Attitude: respondents who scored the mean value and above for attitude-related questions were considered to have a positive attitude and otherwise considered to have a negative attitude [19].

**Data processing and analysis:** the collected data were entered using EPI-data version 4.2.0.0 and analyzed using SPSS version 20. The correlation analysis was done to determine the association between the knowledge sum score and the attitude sum score. A binary logistic regression analysis was done to determine the association between the dependent and independent variables. Independent variables in the bivariate logistic regression model with a p-value of less than 0.25 were included in the multivariable logistic regression. Finally, variables in multivariable logistic regression with a p-value <0.05 were considered as significantly associated with the outcome variable. The Hosmer-Lemeshow goodness-of-fit tests for knowledge (P=0.430) and attitude (P=0.240) showed that the multivariable logistic regression models were good enough to fit the data well.

**Ethical consideration:** ethical approval was obtained from Mizan-Tepi University Institutional Review Board (MTU-IRB) on 12/05/2019 with the reference number MTUIRB/126/2019. Moreover, a support letter was taken from Mizan-Tepi University to Mizan-Aman Health Science College before the actual data collection. All study participants were informed about the purpose of the study, their right to deny participation, anonymity and confidentiality of the information. Written informed consent was also obtained from each participant before participation in the study.

## Results

**Socio-demographic characteristics:** of the 429 students recruited, 394 students have filled the self-administered questionnaire, yielding a response rate of 91.8%. The mean age of the respondents was 22.5 (±3.3 SD) within the range of 18 to 35 years. More than half (53%) of the respondents were females. One hundred sixty-seven (42.3%) and 173 (43.9%) of the respondents were protestant religion followers and in the educational status of secondary and above respectively (Table 1).

**Table 1:** socio-demographic factors of the respondents at MAHSC in Southwest Ethiopia

Variables	Categories	Frequency	Percent
Age	<23 years	176	44.7
	≥23 years	218	55.3
Sex	Male	185	47
	Female	209	53
Religion	Protestant	167	42.3
	Orthodox	120	30.5
	Muslim	107	27.2
Education	No formal education	95	24.1
	Primary school	126	32
	Secondary and above	173	43.9
Residency	Urban	208	52.8
	Rural	186	47.2
Academic year	First-year	121	30.7
	Second-year	137	34.8
	Third-year	136	34.5

**Knowledge and attitude of respondents towards BD:** almost all (95.6%) and more than half (61.2%) of the respondents were aware of BD and were aware through mass media respectively. Three hundred eighty-one (96.7%) knew about their blood group. The majority (90.4%) and more than three-fifth (63.4%) of the respondents were aware of where to donate blood and located blood bank as a place of donation respectively. Generally, more than two-thirds (69.3%) of the respondents had good knowledge of BD (Table 2). Similarly, 58.1% (229) of the respondents had a positive attitude towards BD.

**Table 2:** knowledge related to BD among respondents at MAHSC in Southwest Ethiopia

Variables	Categories	Frequency	Percent
Aware of blood donation (n=394)	Yes	376	95.4
	No	18	4.60
Source of awareness (n=376)	Mass media	230	61.2
	Friends	56	14.9
	Family	47	12.5
	Awareness campaigns	43	11.4
Knowledge of one's blood group (n=394)	Yes	381	96.7
	No	13	3.30
Aware of where to donate blood (n=376)	Yes	340	90.4
	No	36	9.60
Place of blood donation (n=340)	Blood bank	212	62.4
	Health institution	96	28.2
	Blood donation campaigns	32	9.40
Knowledge about blood donation (n=394)	Good	273	69.3
	Poor	121	30.7

**Correlation between knowledge and attitude:** the mean value of knowledge-related questions about blood donation was 7.3 (±1.3 SD) ranges from 3 to 10. The mean value of attitude-related questions towards blood donation was 6.3 (±0.9 SD) ranges from 1 to 7. The knowledge-related questions´ sum score value was positively correlated with the attitude-related questions´ sum score value (r=0.30, P<0.001).

**Factors associated with knowledge and attitude towards BD:** in the bivariate analysis, age group <23 years old, having an educated father, being second or graduating class and ever donated blood were the factors associated with a good knowledge of blood donation at P-value <0.25. Finally, age group <23 years old (AOR=1.67, 95% CI (1.04-2.67)), having a father with primary school (AOR=2.24, 95% CI (1.20-4.17)) and secondary and above school (AOR=2.26, 95% CI (1.26-4.06)) and ever donated blood (AOR=3.64, 95% CI (2.26-5.85)) were the factors associated with good knowledge of blood donation at a P-value <0.05 in the multivariable logistic regression model (Table 3).

**Table 3:** factors associated with good knowledge of BD among respondents at MAHSC in Southwest Ethiopia

Variables	Categories	Knowledge of BD		COR (95% CI)	AOR (95% CI)	P-value
		Poor	Good			
Age group	<23 years	63	104	1	1	
	≥23 years	58	169	1.77(1.15-2.72)**	1.67(1.04-2.67)	0.034
Father education	No formal education	43	52	1	1	
	Primary school	33	93	2.33(1.32-4.11)**	2.24(1.20-4.17)	0.011
	Secondary and above	45	128	2.35(1.39-3.99)**	2.26(1.26-4.06)	0.006
Academic year	First-year	42	79	1	1	
	Second-year	37	100	1.44(0.85-2.44)*	1.61(0.89-2.88)	0.112
	Third-year	42	94	1.19(0.71-2.01)*	1.20(0.67-2.15)	0.538
Ever donated blood	No	83	98	1	1	
	Yes	38	175	3.90(2.47-6.16)**	3.64(2.26-5.85)	<0.001

CI=confidence interval; COR=crude odds ratio; AOR=adjusted odds ratio; * = P-value <0.25; ** = P-value <0.05

In the bivariate analysis, age group <23 years old, female sex, rural resident and being graduating class were the factors associated with a positive attitude towards blood donation at P-value <0.25. Finally, being a rural resident (AOR=1.59, 95% CI (1.01-2.40)) and graduating class student (AOR=0.56, 95% CI (0.34-0.96)) were the factors associated with a positive attitude towards blood donation at a P-value <0.05 in the multivariable logistic regression model (Table 4).

**Table 4:** factors associated with a positive attitude towards BD among respondents at MAHSC in Southwest Ethiopia

Variables	Categories	Attitude towards BD		COR (95% CI)	AOR (95% CI)	P-value
		Negative	Positive			
Age group	<23 years	93	134	0.92(0.61-1.37)*	0.84(0.55-1.28)	0.414
	≥23 years	72	95	1	1	
Sex	Male	76	109	1	1	
	Female	89	120	0.94(0.63-1.40)*	0.88(0.58-1.34)	0.562
Residence	Rural	68	118	1.52(1.01-2.27)**	1.59(1.01-2.40)	0.027
	Urban	97	111	1	1	
Academic year	First-year	42	79	1	1	
	Second-year	60	77	0.68(0.41-1.13)*	0.63(0.37-1.05)	0.076
	Third-year	63	73	0.62(0.37-1.02)*	0.56(0.34-0.96)	0.035

CI=confidence interval; COR=crude odds ratio; AOR=adjusted odds ratio; * = P-value<0.25; ** =P-value <0.05

## Discussion

Several studies suggest that blood donation is a very important medical procedure in saving millions of lives [21,22]. However, BD is affected by several factors, among which people´s knowledge and attitude are the key determinants [10]. A study conducted by Arage *et al*. revealed that good knowledge and a positive attitude towards BD are the determinant factors for the practice of voluntary BD [4]. Based on the above scenario, we conducted a cross-sectional study to assess the level and factors associated with knowledge and attitude towards blood donation among Mizan-Aman health science college students in southwest Ethiopia. As a result, the proportion of good knowledge of blood donation was found to be 69.3%, 95% CI (64.8%-73.4%). This finding was in line with 71% in Riyadh city [20] and 66.7% in Iraq [16]. It was higher than 48.2% and 56.8% in Gondar [13,14], 40.4% in Ambo, Ethiopia [9], 60.2% in Saudi Arabia [6], 54% in Samara [5], 46.43% in Mehal Amba [23], 56.8% in Gondar [14] and 43.5% in Harar, Ethiopia [15] studies in Ethiopia and 60.2% in Saudi Arabia [6]. But it was lower than 82.6% in Woliyta Sodo, Ethiopia [7] and 97.1% in Malaysia [17].

The proportion of positive attitude towards BD was found to be 58.1%, 95% CI (53.2%-63.0%). This finding was in line with 58.7% in Woliyta Sodo, Ethiopia [7]. It was higher than 47.4% in Ambo, Ethiopia [9], and 32.9% in Harar [15] studies in Ethiopia. But it was lower than 65.8% in Samara [5], 79.2% and 82% in Gondar [13,14] studies in Ethiopia, 71% in Riyadh city [20], 68.7% in Iraq [16], and 88.8% in Malaysia [17]. The variation observed compared to other studies could be due to the differences in methodology, sample size and operational definitions used. Besides, our study participants are health science students which will have a positive perception of blood donation [24] and this may create a significant variation.

Of the 273 respondents with good knowledge of BD, 169 (61.9%) were in the age category of 23 years and above. Respondents with the age category of 23 years and above had 1.7 times increased odds of having good knowledge of BD compared to age less than 23 years. Age was statistically associated with knowledge of BD. This finding was consistent with a study conducted in Harar, Ethiopia [15]. But this was contrary to a study done in Riyadh city, which revealed that young age is more likely to have good knowledge than older age [20]. Respondents who had a father with primary and secondary and above had 2.2 and 2.3 times increased odds of having good knowledge of BD respectively compared to respondents whose fathers had no formal education. This finding was in-line with a study conducted in Ambo, Ethiopia [9]. This could be because educated fathers are more likely to be aware of their children and family as a whole.

Of the 273 respondents with good knowledge of BD, 175 (64.1%) ever donated blood. Respondents who practiced blood donation had 3.6 times increased odds of having good knowledge about BD than those who did not donate. This finding was consistent with a study conducted in Kerman city [25]. But this was inconsistent with a study conducted in Harar, which revealed that the proportion of study participants who donated blood voluntarily and had good knowledge about voluntary blood donation was significantly lower than the study participants who donated blood voluntarily and had a low knowledge [15]. Of the 229 respondents with a positive attitude towards BD, 73 (31.9%) were third-year students (graduating class students). Respondents with third-year students (graduating class students) were 44% less likely to have a positive attitude towards BD than with first and second-year students. A study done by Gebresilase *et al*. [19] supported this finding. But, it was inconsistent with a study conducted in Ambo, Ethiopia [9].

Students from rural residence were approximately 1.6 times more likely to have a positive attitude than students from urban areas. This could be explained by the rural community is more likely to feel and be sensitive for a person with an injury that needs blood, which makes them develop a better positive attitude towards BD. The knowledge sum score was correlated with the attitude sum score (r=0.30, P<0.001). This shows that there is a positive fair degree of relationship between knowledge and attitude. The authors acknowledge, nature of the study (cross-sectional study) may not show the cause-and-effect relationship and the social desirability bias of the respondents may overestimate or underestimate the results of this study.

## Conclusion

The knowledge and attitude towards BD among the study population are a substantial deficiency. Therefore, more effort is needed to increase the level of knowledge and attitude towards BD by inculcating short training courses for these groups of population in the existing curriculum.

### What is known about this topic

- Each year millions of lives are saved through blood transfusions, however, in most developing countries, including Ethiopia, people still die due to a shortage of blood and blood products;
- The World Health Organization (WHO) advocates that 3-5% of the country´s population should donate blood yearly to meet the needs of blood and blood products;
- The process of blood donation (BD) is affected by several factors, among which people´s level of knowledge and attitude are the key determinants.

### What this study adds

- The proportions of good knowledge and positive attitude towards BD were 69.3% and 58.1% respectively;
- The knowledge-related questions´ sum score value was positively correlated with the attitude-related questions´ sum score value;
- Younger age, having an educated father and ever donated blood were factors associated with good knowledge of BD; being a rural resident and graduating class student were the factors associated with a positive attitude towards BD.
